# 349. Diagnostic Testing and Antibiotic Utilization in Patients with COVID-19

**DOI:** 10.1093/ofid/ofab466.550

**Published:** 2021-12-04

**Authors:** Lauren Groft, Iulia Opran, Yeabsera Tadesse, Hang Vo, Emily Heil, Emily Heil, Gregory Schrank, Kimberly C Claeys

**Affiliations:** 1 The Johns Hopkins Hospital, Baltimore, MD; 2 University of Maryland School of Pharmacy, Baltimore, MD; 3 University of Maryland School of Pharmacy; University of Maryland Medical Center, Baltimore, MD; 4 R Adams Cowley Shock Trauma Center, Baltimore, MD

## Abstract

**Background:**

Patients with COVID-19 receive high rates of antibiotic therapy, despite viral origin of infection. Reports of bacterial coinfection range from 3.5 to 8% in the early phase of infection. This study aimed to evaluate the relationship between diagnostic tests and antibiotic utilization in patients admitted with COVID-19 at the University of Maryland Medical Center to better inform future prescribing practices.

**Methods:**

Retrospective cohort study of adult patients with a positive SARS-CoV-2 PCR on admission from March 2020 through June 2020. Associations between diagnostic tests employed and antibiotic initiation and duration were explored using bivariate analysis (SPSS®).

**Results:**

Baseline characteristics of 224 included patients are reported in Table 1. Excluding SARS-CoV-2 PCRs, most frequently performed diagnostic tests included blood cultures (65.6%), MRSA nasal surveillance (45.1%), respiratory cultures (36.2%), respiratory viral panel (RVP) (33.0%), and Legionella (28.6%) and pneumococcal (26.3%) urine antigens. Positivity of RVP, Legionella, pneumococcus, blood, and respiratory tests were low (1.3%, 0.4%, 0.9%, 1.8%, and 6.7%, respectively). A total of 62% of patients were initiated on antibacterial therapy with a median cumulative antibiotic duration of 77.9 hours (IQR 41.4, 111.8). History of chronic respiratory disease (76% vs. 58.6%; *P*=0.025), any degree of oxygen requirement on admission (72% vs. 42.6%; *P*=0.006), and performance of blood cultures (70.7% vs. 46.8%, *P*< 0.0001) were associated with antibiotic initiation. Positive bacterial diagnostic respiratory culture (median duration 72.8h [IQR 46.7, 96.6] vs. 97.3h [IQR 79.8, 194.1]; *P*=0.027) and positive blood culture (median duration 80.1h [IQR 42.1, 111.7] vs. 97.5h [IQR 71.8, 164.8]; *P*=0.046) were associated with longer antibiotic duration. Patients who did not have respiratory cultures performed had similar antibiotic durations as those with negative respiratory cultures.

Table 1. Baseline Characteristics

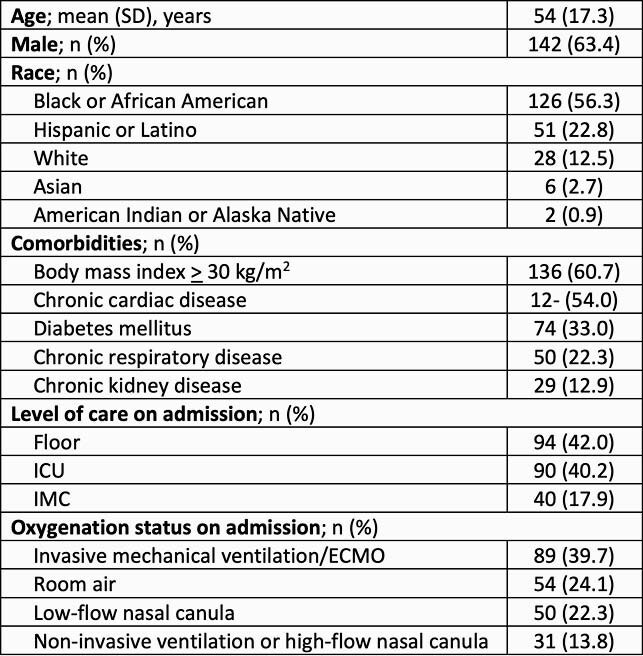

**Conclusion:**

Despite low coinfection rates, negative diagnostic tests did not result in shorter empiric antibacterial duration. These findings highlight the ongoing need for both diagnostic and antimicrobial stewardship in COVID-19.

**Disclosures:**

**Emily Heil, PharmD, MS, BCIDP**, Nothing to disclose **Kimberly C. Claeys, PharmD**, **GenMark** (Speaker’s Bureau)

